# Supplementary material for: Empirical methods for controlling false positives and estimating confidence in ChIP-Seq peaks
Source: BMC Bioinformatics. 2008 Dec 5;9:523. doi: 10.1186/1471-2105-9-523 (PMC2628906; doi:10.1186/1471-2105-9-523)
Supplement: Additional File 1 — A variety of html documents from the USeq web site detailing the available applications, their best usage, output file type descriptions, command line menus, etc. [file 1471-2105-9-523-S1.zip › USeqUserGuides/JavaDoc.zip!/JavaDoc/bioroot/plasmid/package-summary.html]

bioroot.plasmid


|  |  |  |
| --- | --- | --- |
| **Overview** \| **Package** \| Class \| **Use** \| **Tree** \| **Deprecated** \| **Index** \| **Help** | |  |
| **PREV PACKAGE**   **NEXT PACKAGE** | **FRAMES**    **NO FRAMES**     **All Classes** |  |


---

## Package bioroot.plasmid

| **Class Summary** | |
| --- | --- |
| **NewPlasmid** |  |
| **OligoMatch** |  |
| **PlasmidBase** |  |
| **PlasmidBean** |  |
| **PlasmidModify** | Servlet to modify plasmids using PlasmidBeans referenced from a Session object. |
| **PlasmidSpreadSheet** |  |
| **PlasmidUpload** |  |
| **PlasmidUploadBean** | Plasmid bulk file upload bean. |
| **PlasmidViewPreferences** |  |

---


|  |  |  |
| --- | --- | --- |
| **Overview** \| **Package** \| Class \| **Use** \| **Tree** \| **Deprecated** \| **Index** \| **Help** | |  |
| **PREV PACKAGE**   **NEXT PACKAGE** | **FRAMES**    **NO FRAMES**     **All Classes** |  |


---
